# Absence of lysogeny in wild populations of *Erwinia amylovora* and Pantoea agglomerans

**DOI:** 10.1111/1751-7915.12253

**Published:** 2015-02-12

**Authors:** Dwayne R Roach, David R Sjaarda, Calvin P Sjaarda, Carlos Juarez Ayala, Brittany Howcroft, Alan J Castle, Antonet M Svircev

**Affiliations:** 1 Department of Biological Science, Brock University500 Glenridge Avenue, St. Catharines, ON, L2S 3A1, Canada; 2 Agriculture and Agri-Food Canada4902 Victoria Ave. North, P.O. Box 6000, Vineland Station, ON, L0R 2E0, Canada

## Abstract

Lytic bacteriophages are in development as biological control agents for the prevention of fire blight disease caused by *Erwinia amylovora*. Temperate phages should be excluded as biologicals since lysogeny produces the dual risks of host resistance to phage attack and the transduction of virulence determinants between bacteria. The extent of lysogeny was estimated in wild populations of *E. amylovora* and *Pantoea agglomerans* with real–time polymerase chain reaction primers developed to detect *E. amylovora* phages belonging to the *Myoviridae* and *Podoviridae* families. *Pantoea agglomerans*, an orchard epiphyte, is easily infected by *Erwinia* spp. phages, and it serves as a carrier in the development of the phage-mediated biological control agent. Screening of 161 *E. amylovora* isolates from 16 distinct geographical areas in North America, Europe, North Africa and New Zealand and 82 *P. agglomerans* isolates from southern Ontario, Canada showed that none possessed prophage. Unstable phage resistant clones or lysogens were produced under laboratory conditions. Additionally, a stable lysogen was recovered from infection of bacterial isolate Ea110R with *Podoviridae* phage ΦEa35-20. These laboratory observations suggested that while lysogeny is possible in *E. amylovora*, it is rare or absent in natural populations, and there is a minimal risk associated with lysogenic conversion and transduction by *Erwinia* spp. phages.

## Introduction

*Erwinia amylovora,* the causative agent of fire blight, is a highly challenging bacterial plant pathogen that threatens sustainable pome fruit production in North America, Central Europe, Middle East and New Zealand (Van der Zwet *et al*., 2012). The disease affects many species in the *Rosaceae* family, and in particular the economically important apple (*Malus* X *domestica*) and pear (*Pyrus communis*) cultivars (Vanneste, 2000; Van der Zwet *et al*., 2012). Bacterial colonization of the flower pistil and hypanthium leads to subsequent migration into plant tissues. The collapse of the plant parenchyma causes the typical disease symptoms of necrosis, wilting and ooze production. Depending upon the climatic conditions, fire blight outbreaks may destroy entire trees and orchards within a single season. Historically, control measures relied on the use of the highly efficacious antibiotic streptomycin (Stockwell and Duffy, 2012). The development of streptomycin resistance (McManus *et al*., 2002; Russo *et al*., 2008), and the regulatory restrictions on the use of antibiotics in agriculture have resulted in a move away from antibiotic use in certain geographic regions (Johnson and Temple, 2013). Biological control agents (BCAs) or biologicals were developed and made available for the control of fire blight in commercial orchards (Stockwell *et al*., 2009; Sundin *et al*., 2009; Kabaluk *et al*., 2010; Pusey and Stockwell, 2011). Biologicals are typically incorporated into integrated pest management programs and in some regions used as a supplement to the more conventional streptomycin applications. Commercially available biologicals have a common mode of action that involves pathogen suppression by providing a barrier, produce antibiotic compounds and/ or a nutrient competition with the pathogen (Stockwell *et al*., 2002; Pusey *et al*., 2009; Sundin *et al*., 2009).

Bacteriophages (phages) offer a novel biological control mechanism since they are ubiquitous in the orchard environment, self-replicating, nontoxic to eukaryotes, biodegradable unlike many agrochemicals and usually species and strain specific with no effect on indigenous bacteria. Phage-mediated control of fire blight has been studied in laboratory and under field conditions (Erskine, 1973; Gill *et al*., 2008; Lehman, 2007; Müller *et al*., 2011; Svircev *et al*., 2010; 2011; Boulé *et al*., 2011; Schwarczinger *et al*., 2011; Nagy *et al*., 2012). Erskine (1973) first demonstrated that a temperate phage that lysogenized a yellow saprophytic bacterium reduced fire blight symptoms when co-inoculated with *E. amylovora* on pear slices. This saprophytic bacterium delivered the temperate phage and was independently antagonist to *E. amylovora*. Subsequently, field-based trials and *in planta* bioassays have demonstrated that *Erwinia* spp. phages have the ability to control *E. amylovora* at levels comparable to that of streptomycin (Lehman, 2007; Svircev *et al*., 2010; 2011). This control was achieved by applying lytic phages along with a non-pathogenic orchard bacterial epiphyte, *Pantoea agglomerans*. *Pantoea agglomerans* plays a dual role in this system. Primarily, it provides sacrificial host cells (carriers) that propagate the lytic phage population that otherwise would succumb to desiccation or UV degradation on the blossom surface. Second, *P. agglomerans* is also a biological control agent that colonizes the flower pistil and prevents further colonization by *E. amylovora* (Lehman, 2007).

A major risk of phage therapy is associated with the inadvertent use of temperate phages, which can enter the lysogenic state by integration into the host genome and exist as a prophage. Lysogens, bacterial hosts with prophage(s), cannot be infected by homologous phage through superinfection immunity (Abedon, 2008). This phage resistance mechanism produces a population of resistant bacteria, which may negate BCA efficacy. The presence of a prophage may also increase the potential of transduction, phage-mediated transfer of genes that can improve bacterial virulence and/or fitness (Wagner and Waldor, 2002; Brüssow *et al*., 2004; Canchaya *et al*., 2004; Brüssow, 2009; Addy *et al*., 2012b), contribute to loss of phytopathogen virulence (Addy *et al*., 2012a), change host cell morphology (Weinbauer, 2004; Addy *et al*., 2012a,b), transfer toxin genes (Mauro and Koudelka, 2011), impact strain individuality (Canchaya *et al*., 2003; 2004), alter sensitivity to antibiotics (Wagner and Waldor, 2002), spread antibiotic resistance (Muniesa *et al*., 2013), activate restriction-methylation systems (Weinbauer, 2004) and improve bacterial survival under poor growth conditions (Williamson *et al*., 2002; Williamson and Paul, 2006; Paul, 2008).

Temperate phages have been found in many phytopathogens and plant-associated bacteria such as *Rhizobium* spp*.* (Malek, 1990; Uchiumi *et al*., 1998), *Ralstonia solanacearum* (Yamada *et al*., 2007; Murugaiyan *et al*., 2011; Addy *et al*., 2012a,b), rhizosphere pseudomonads (Shaburova *et al*., 2000), *Xanthomonas campestris* pv. *azadirachtae* (Borkar, 1997), *Erwinia carotovora* subsp. *carotovora* (Faltus and Kishko, 1980; Ruban *et al*., 1981; Toth *et al*., 1993; Tovkach, 2002a,b) and *P. agglomerans* (previously known as *Erwinia herbicola*) (Harrison and Gibbins, 1975). Furthermore, the genomes of all fully sequenced *E. amylovora* phages lack the necessary genes for prophage integration (Lehman *et al*., 2009; Born *et al*., 2011; Nagy *et al*., 2012).

The main purpose of this study was to screen with real-time polymerase chain reaction (PCR) for the occurrence of lysogeny in *E. amylovora* wild-type isolates obtained from a global collection. Screening for prophages was extended to the orchard epiphyte *P. agglomerans* since research showed that *P. agglomerans* isolates, *Pantoea vegans* C9-1, formerly known as *Pantoea agglomerans* C9-1 and *P. agglomerans* E325 (Svircev unpublished data) can be easily infected by *Erwinia* spp. phages (Lehman, 2007). In addition, classical induction methods were used to produce bacteriophage insensitive bacterial mutants (BIMs) by challenging the host with high-titre phage solutions. Collectively, this research facilitated in the understanding of lysogeny in *E. amylovora* and its bacteriophages. This information will help designate which lytic phages should be selected as components of phage-carrier mixture in the development of this novel biological control system.

## Results

### Real-time PCR screening for prophages

Multiplex real-time PCR was used to detect the presence of prophage DNA in *E. amylovora* (Table 1, Table S1) and *P. agglomerans* (Table 1, Table S2) isolates collected from various rosaceous hosts. All 161 *E. amylovora* isolates originating from 16 global locations were identified as *E. amylovora* with Ea-*lsc* primers/probe targeting the levansucrase gene (Table 2). Eighty-two isolates of *P. agglomerans* were collected from pear, apple, mountain ash, crab apple, hawthorn and cotoneaster blossom washings. *Pantoea agglomerans* identity was confirmed with Pa-*gnd*2 primers and probe targeting the gluconate-6-dehydrogenase gene (Table 2). Specificities for the *Podoviridae* and *Myoviridae* phage primer/probe combinations were confirmed by screening numerous phage isolates (Table 3) that had been previously identified with electron microscopy and restriction fragment length polymorphism (RFLP) analysis (Gill *et al*., 2008). All isolates of *E. amylovora* and *P. agglomerans* were shown to be negative for both phage prophages families (Table 4) regardless of the geographic location of the isolation site. In addition, seven isolates of *Erwinia pyrifolia* from Korea and Japan were tested similarly, and no evidence of prophages was obtained (data not shown).

**Table 1:** Sources of wild-type bacteria isolates and bacteriophages

Isolate	Source/Phage family	Reference
*E. amylovora*		
Ea6-4	*Malus* X *domestica*	A.M. Svircev
EaD-7	*Malus* X *domestica*	A.M. Svircev
Ea29-7	*Malus* X *domestica*	A.M. Svircev
EaG-5	*Pyrus communis*	(Gill *et al*., 2008)
Ea17-1-1	*Malus* X *domestica*	(Gill *et al*., 2008)
Ea110R	*Malus* X *domestica*	(Gill *et al*., 2008)
155 Isolates	Global Collection, AAFC Vineland	Table S1
*P. agglomerans*		
Pa1	*Malus* X *domestica*	This study
Pa1-28a	*Malus* X *domestica*	″
Pa1-28b	*Malus* X *domestica*	″
Pa7-5	*Malus* X *domestica*	″
Pa17-17	*Malus* X *domestica*	″
Pa21-5	*Pyrus communis*	″
76 Isolates	*Malus* and *Pyrus*	Table S2
*Erinia* spp. bacteriophages		
ΦEa10-1	*Myoviridae*	(Gill *et al*., 2008)
ΦEa10-2	*Myoviridae*	″
ΦEa10-4	*Myoviridae*	″
ΦEa21-2	*Myoviridae*	″
ΦEa21-4	*Myoviridae*	″
ΦEa21-3	*Myoviridae*	″
ΦEa31-1	*Myoviridae*	″
ΦEa35-2	*Myoviridae*	″
ΦEa45-1B	*Myoviridae*	″
ΦEa9-2	*Podoviridae*	(Gill *et al*., 2008)
ΦEa9-4	*Podoviridae*	″
ΦEa10-7	*Podoviridae*	″
ΦEa10-8	*Podoviridae*	″
ΦEa10-9	*Podoviridae*	″
ΦEa10-10	*Podoviridae*	″
ΦEa10-11	*Podoviridae*	″
ΦEa10-13	*Podoviridae*	″
ΦEa10-15	*Podoviridae*	″
ΦEa31-3	*Podoviridae*	″
ΦEa35-20	*Podoviridae*	This study
ΦEa35-6	*Podoviridae*	″
ΦEa46-1A2	*Podoviridae*	″
ΦEa51-2	*Podoviridae*	(Gill *et al*., 2008)
ΦEa51-4	*Podoviridae*	″
ΦEa51-6	*Podoviridae*	
ΦEa51-7	*Podoviridae*	″

**Table 2:** Primers and TaqMan probes utilized in multiplex real-time PCR

Target/Primer name^a^	Sequence (5′ – 3′)^b^	Amplicon size (nt)	Source
*E. amylovora*			
Ea-Lsc-F	cgctaacagcagatcgca	105 bp	(Lehman, 2007)
Ea-Lsc-R	aaatacgcgcacgaccat		
Ea-Lsc-P	CY5/ctgataatccgcaattccaggatg\IAbRQSp		
*P. agglomerans*			
Pa-Gnd2-F	cgctaacccgactgtgct	73 bp	This study
Pa-Gnd2-R	tgaaggtttgccctttgc		
Pa-Gnd2-P	FAM/atgacaccatcatcgtaaaggcgg\BHQ_1		
*Myoviridae*			
PUN45-F	aacgaacagcgccttgac	140 bp	This study
PUN45-R	ccagttgcagccagtgtg		
PUN45-P	ROX/actgagaagtacggtatcaaaccttc/IAbRQS		
*Podoviridae*			
STS3-F	gacaaacaagaacgcggcaactga	96 bp	This study
STS3-R	atacccagcaaggcgtcaacctta		
STS3-P	FAM/agatgaagtaggttatcttcacagtgccct/BHQ_1		

a F forward primer, R reverse primer, P probe.

b Flurorophores: Cy5, 6-FAM, HEX, ROX; Quenchers: IAbRQSp NHS Ester Iowa Black RQTM-Sp, BHQ_1 Black Hole Quencher. Integrated DNA Technologies, Coralville, IA, USA.

**Table 3:** Summary of real-time PCR diagnostics using *Podoviridae* and *Myoviridae* specific probe and primers

Bacteriophage Family	Probe/Primer	
	STS3	PUN45
*Podoviridae*	16/17^a^	0/17
*Myoviridae*	0/9	9/9

a Number of phages detected/total number of phages. Phage family was determined by RFLP and TEM (Gill *et al*., 2008) and probe/primer detection (this paper). Only *Erwinia* spp. phage Φ9-2 was not detected by *Podoviridae*-specific probe/primer.

**Table 4:** Summary of screening results for the presence of lysogeny in wild-type global populations of *E. amylovora* and *P. agglomerans* using real-time PCR

Isolate^a^	Location	Isolates tested	*Lysogeny*^b^	
			*Myoviridae*	*Podoviridae*
*E. amylovora*	ON, Canada	5	−	−
	BC, Canada	10	−	−
	NS, Canada	10	−	−
	CA, USA	14	−	−
	MI, USA	16	−	−
	NY, USA	11	−	−
	OR, USA	12	−	−
	France	10	−	−
	Germany	10	−	−
	Israel	10	−	−
	Lebanon	12	−	−
	Morocco	10	−	−
	New Zealand	6	−	−
	Poland	12	−	−
	Spain	9	−	−
	Switzerland	4	−	−
*P. agglomerans*	ON, Canada	82	−	−

a Tables S1 and S2 provide a detailed list of wild-type isolates and their sources.

b Presence of prophage tested using real-time PCR, utilizing STS3 (*Podoviridae*) and Pun45 (*Myoviridae*) probe/primer sets − indicates a negative result.

### Screening for prophages: spontaneous release and mitomycin C induction

Evidence of lysogeny was not obtained from screening cultures for the spontaneous release of phage progeny nor the induction of prophage release through recombination. Supernatants of liquid cultures of *P. agglomerans* failed to produce any plaques when added to *E. amylovora* indicator isolates Ea6-4, Ea17-1-1, EaG-5, Ea110R and Ea29-7 (Table S2). Mitomycin C at 0.5 or 1 μg/ml also failed to induce any phages in a subgroup of 22 *P. agglomerans*, and no plaques were observed on the same indicator isolates (data not shown).

### Recovery of bacteriophage insensitive mutants

After numerous trials using the phage challenge methods and various combinations of phages and hosts (data not shown), the secondary culture method was more efficient at isolating BIMs than the agar overlay method. Several BIMs originating from sensitive parental isolates were recovered from *E. amylovora* Ea29-7, Ea17-1-1, G-5 and Ea110R. All BIMs were confirmed by real-time PCR as *E. amylovora*. However, *Podoviridae* and *Myoviridae* DNAs were not detected in BIMs derived from Ea29-7, Ea17-1-1 and G-5. These BIMs were deemed to lack a prophage and were not tested further.

Initially, 42 BIMs were recovered from Ea110R after challenge with ΦEa35-20 (*Podoviridae*). After four rounds of sub-culturing, 24 survivors maintained their phage resistance. To determine the stability of lysogeny, BIMs were cultured from frozen stocks following 2, 6, 12 and 24 months of storage and monitored by real-time PCR for the conservation of *Podoviridae* phage DNA. Of the 24 BIMs, 12 were unstable and showed a loss or decrease in prophage signal over the course of the trials. Twelve BIMs remained stable and produced similar C_T_ values in most trials. These lysogens maintained an expected approximate C_T_ ratio of 1:1 between *Podoviridae* and *E. amylovora* sequences (Table 5). After 24 months in storage at −80°C, 22 of the BIMs were tested for stability. Eleven BIMs were previously identified as unstable and 11 as stable. Ten colonies recovered from each BIM were analysed separately for the presence of phage DNA. Only three of the formerly stable BIMs remained stable with 8 to 10 of the colonies showing a 1:1 phage to bacterial DNA ratio (Table 5).

**Table 5:** Dualplex real-time PCR of isolated *E. amylovora* Ea110R bacteriophage insensitive mutants (BIMs)

BIM	Sample 1^b^		Sample 2^c^		Sample 3^d^		Sample 4^e^
	*Ea* aC_T_	Phage C_T_	*Ea* C_T_	Phage C_T_	*Ea* C_T_	Phage C_T_	1:1 ratio
1	18.3	32.8	17.2	>40	20.5	>40	0/10
6	15.9	32.8	20.9	37.8	19.6	>40	0/10
7	17.0	36.0	19.5	>40	17.4	>40	ND
8	17.2	18.7	17.1	>40	17.4	19.2	10/10
10	15.4	19.6	16.4	11.6	18.5	13.9	4/10
16	16.0	35.3	18.2	39.2	17.1	>40	1/10
18	16.5	16.6	17.9	12.4	16.9	13.1	10/10
19	17.6	18.1	19.6	17.3	19.0	16.8	ND
21	15.8	22.2	17.0	>40	18.1	>40	0/10
22	15.3	33.3	19.0	>40	17.3	37.7	0/10
23	17.2	30.3	17.2	>40	17.5	>40	7/10
24	16.2	17.0	20.5	25.9	18.5	24.8	0/10
25	16.3	19.7	16.4	39.8	19.3	>40	1/10
26	15.5	15.3	16.3	14.9	19.6	21.8	3/10
27	16.9	14.6	15.9	13.1	16.6	16.6	0/10
29	16.2	31.5	18.6	>40	16.7	>40	0/10
34	15.5	26.1	18.9	14.8	20.2	16.5	9/10
35	16.5	30.6	19.8	13.7	19.2	13.6	6/10
37	17.0	32.6	18.1	>40	20.8	37.2	0/10
38	17.2	29.6	19.6	37.0	18.5	>40	1/10
39	16.7	16.3	17.5	9.4	18.2	15.3	6/10
40	16.1	16.8	18.6	13.0	16.8	13.2	0/10
41	16.8	20.9	17.3	16.7	20.2	21.5	0/10
42	17.1	29.4	17.6	37.6	16.8	>40	0/10

a Threshold cycle (C_T_) values were derived from purified Ea110R BIM colonies by amplifying *E. amylovora* DNA with *Lsc* primer/probes (*Ea* C_T_) and *Podoviridae* phage DNA with *STS*3 primers/probe (Phage C_T_): Single colonies were recovered from frozen stocks at three times:

b Sample 1, at 2 months post-storage at −80°C;

c Sample 2, at 6 months post-cold storage;

d Sample 3, at 12 months post-cold storage.

e Sample 4, at 24 months of storage where 10 colonies from each BIM stock were analysed, and the number of colonies that showed similar C_T_ values for *E. amylovora* and phage DNA were reported.

### Recovery of a temperate phage

Treatment of BIM 8 (Table 5) with ultraviolet light resulted in the recovery of a viable phage, presumably a clone of ΦEa35-20, belonging to the *Podoviridae*. Restriction fragment length polymorphism analysis with *Bam*H1, *Eco*R1 and *Bgl*II showed similar fragment patterning to known *Erwinia* spp. *Podoviridae* phages (Roach, 2011) and the genome size was estimated to be at 46 000 bp (data not shown). To assess any altered host range due to lysogeny, BIM 8 was challenged with *Podoviridae* phages ΦEa31-3 and ΦEa46-1A2 and *Myoviridae* phage ΦEa45-1B. This BIM was resistant to lysis by ΦEa31-3 and ΦEa46-1A2 but remained susceptible to ΦEa45-1B.

## Discussion

Phages represent the most abundant biological form in the biosphere with an estimated total number of 10^31^ particles, outnumbering host bacteria 10-fold (Brüssow *et al*., 2004). Not surprisingly, temperate phages are ecologically important, shaping bacterial communities by controlling their composition, numbers and activities. Comparative genomics have revealed the pervasiveness of prophages in most bacterial genomes and constitute one of the main sources of genetic diversity and strain variation. They are associated with promoting host immunity, virulence and contribution to the evolution of several important bacterial pathogens (Bondy-Denomy and Davidson, 2014). We anticipated that they would play similar roles in *E. amylovora* and *P. agglomerans* populations*.* However, screening of 161 *E. amylovora* isolates from 16 distinct geographic areas worldwide failed to show evidence of any isolate possessing *Myoviridae* or *Podoviridae* prophages. Similarly, a pan-genome study of 12 strains of *E. amylovora* identified trace phage remnants but no intact prophages (Mann *et al*., 2013). Nonetheless, phage ΦEa35-20 was clearly identified as a temperate phage of *E. amylovora* 110R in the present work. The Ea110R lysogen carrying prophage ΦEa35-20 was immune to two *Podoviridae* phages but remained susceptible to a *Myoviridae* phage. The lysogen therefore demonstrated homo-infection immunity to similar phages.

Prophages were also not observed in a genomic screening of 82 *P. agglomerans* isolates from rosaceous hosts in southern Ontario, Canada even though lysogeny has been previously reported in this species (Erskine, 1973; Harrison and Gibbins, 1975). Further testing of a subgroup of *E. amylovora* and *P. agglomerans* did not show any signs of release of phage particles spontaneously or after lytic cycle induction attempts with mitomycin C. These results indicate that lysogeny in *E amylovora* and *P. agglomerans*, although possible, is extremely rare and/or absent.

The lack of lysogeny in *E. amylovora* and *P. agglomerans* reported here contests the overall consensus that lysogeny is a prevalent life choice by most Gram negative bacteria (Ackermann and Dubow, 1987; Jiang and Paul, 1998; Williamson *et al*., 2002; 2007; 2008a,b; Brüssow *et al*., 2004; Canchaya *et al*., 2004; Ghosh *et al*., 2008; Swanson *et al*., 2012). Canchaya and colleagues (2004) acknowledge that prophages may be absent from a genome since prophages can be easily acquired and lost from the bacterial cell. While little is known about the factors leading to the establishment of different lifestyles, the choice is believed to be determined to a great extent by the metabolic health of the host bacteria. The lysogenic cycle may be undertaken when host abundance is low and conditions for replication are unfavourable (Marsh and Wellington, 1994; Weinbauer, 2004). Extreme and low nutrient environments have been shown to favour phages that readily undertake a lysogenic life cycle (Jiang and Paul, 1998; Williamson *et al*., 2002; Williamson, 2008a,b). Blossom stigmatic surfaces provide a nutrient-rich environment for the pathogen and epiphyte (Johnson and Stockwell, 1998; Pusey, 2000; Pusey and Smith, 2008; Stockwell *et al*., 2008; Pusey *et al*., 2009). This habitat does not include harsh environmental conditions or low host abundance necessary to promote lysogeny. The bacteria that were studied here were likely in a state of vigorous growth due to ideal nutrient and environmental conditions provided by the plant. Because of this, it is plausible that lytic lifestyles were favoured leading to an absence of lysogeny. This argument applies equally to *E. amylovora* and *P. agglomerans*. *Erwinia* spp. phages are known to be able to cross the species barrier and infect the orchard epiphyte *P. agglomerans* (Erskine, 1973; Lehman, 2007; Svircev *et al*., 2010; 2011)*.* Independently, Erskine (1973) and Harrison and Gibbins (1975) reported that phages that could infect *E. amylovora* were capable of a lysogeny in *P. agglomerans*. In the present study, no evidence of lysogeny was apparent in *P. agglomerans*, which suggested that this bacterium does not provide a reservoir for *Erwinia* spp. phages during unfavourable growth conditions. To achieve a broader understanding into the incidence of lysogeny in *E. amylovora* and *P. agglomerans*, sampling of more variable environments will give a deeper understanding of phage–host relationship in natural conditions (Brüssow, 2012) and may reveal higher frequencies of lysogeny (Williamson *et al*., 2007; 2008a,b) as well as greater genotypic and phenotypic diversity in orchard pathogen and epiphyte communities.

The advent of real-time PCR has allowed for an efficient and accurate detection of prophages in many Gram-negative and Gram-positive bacteria (Lunde *et al*., 2000; 2003; Sobéron *et al*., 2007; del Rio *et al*., 2008; Pecson *et al*., 2009; Rodriguez *et al*., 2009). Multiplex real-time PCR was used in this study to simultaneously analyse *E. amylovora* or *P. agglomerans* isolates for the presence of *Myoviridae* and *Podoviridae* prophage within a single sample. The extreme sensitivity of real-time PCR enabled us to track the loss of prophage DNA from *E. amylovora* BIMs that were originally identified as lysogens. During lysogeny, a prophage is integrated into the host genome resulting in an approximate C_T_ ratio of 1:1 of phage : host values. Most of the BIMs had phage : host ratios that changed over time even though a 1:1 ratio was initially apparent. Phage DNAs were not stable, and presumptive lysogens experienced the eventual loss or significant reduction of prophage copy number. This variation in stability could be the result of prophage eviction without loss of bacterial cell viability by mechanisms that are very unclear (Weinbauer, 2004; Khemayan *et al*., 2006; Abedon, 2008).

A more plausible explanation is the unstable coexistence of a phage genome in the host bacterium, where that genome fails to replicate either as a productive infection or with cell division as seen with pseudolysogenic lifestyles (Williamson *et al*., 2001; Khemayan *et al*., 2006; Łoś and Węgrzyn, 2012). Pseudolysogeny is often characterized by high host cell and phage copy numbers in a bacterial culture but the lytic cycle cannot be induced by mitomycin C (Williamson *et al*., 2001; Pasharawipas *et al*., 2008). Properties such as superinfection immunity are nonetheless exhibited by the host (Ripp and Miller, 1998). Khemayan *et al*. (2006) reported a similar event where *Vibrio harveyi* presumptive lysogen cell stability varied widely, and numerous cultures constantly generated large numbers of cured cells that had lost their lysogenic status. The presence of unstable lysogens and/or pseudolysogens has been observed in other bacterial species such as *Listonella pelagia* (Williamson *et al*., 2001). Further work on the BIMs of *E. amylovora* is required for the elucidation of the mechanism of the lack of stability.

To the best of our knowledge, this is the first study that examines the incidence of lysogeny in an orchard phytopathogen and epiphytic bacterium. This study was facilitated by the use of a simple and rapid molecular-based multiplex real-time PCR assay which detected prophages in their quiescent state, circumventing the laborious and inaccurate induction-dependent techniques. Lysogeny was demonstrated for *E. amylovora* under laboratory conditions and for *P. agglomerans* in an orchard isolate (Erskine, 1973). Lysogeny appears to be rare or absent when bacteria reside in the phyllosphere and are isolated from nutrient-rich environments such as flower washings. These observations indicate that the risks associated with transduction and lysogenic conversion from the use of phages and the carrier host to control fire blight during bloom may be low. Greater reduction of risk would be achieved by the use of a mixture or cocktail of several different lytic phages in the biological control agent. This mixture should minimize the risk of development of bacterial resistance and broaden the range of hosts controlled by the agent, as has been shown for phage cocktails against diarrhoea-associated *E. coli*. (Bourdin *et al*., 2014). Further optimization of the phage-based biological would involve selection of phages for the cocktails based on host range studies and the selection of a carrier isolate of *P. agglomerans* that allows the production of high-phage titres and secondary antimicrobial metabolites.

## Experimental procedures

### Bacteria and bacteriophages

Bacteria and bacteriophages are listed in Table 1. Modified Miller-Schroth Medium [MMS; 0.8% nutrient broth (NB, Difco Laboratories, Sparks, MD), 2.5% sucrose, 2% Bacto agar (Difco), 0.0045% bromothymol blue, 0.00125% neutral red, pH 7.4] was used initially to recover *E. amylovora* and *P. agglomerans* isolates from apple and pear orchards (Brulez and Zeller, 1981). Bacterial stocks were stored at −80°C in 0.8% NB, 0.25% yeast extract (Difco), 0.5% sucrose, 0.25% K_2_HPO_4_, 0.05% KH_2_PO_4_, 0.025% MgSO_4_ and 50% (v/v) glycerol. Bacteria were cultured from frozen stocks on 2.3% nutrient agar (NA; Difco) or 0.8% NB, 0.25% yeast extract (YE) and 0.5% sucrose and incubated at 27°C for 18–48 h prior to use. Phages were enriched by inoculating 25 ml of 0.8% NB with 100 μl of bacterial host at 0.6 at OD_600_ (∼ 10^8^cfu/ml) and 1 ml phage stock solution at 10^8^ pfu/ml, at 27°C for 20 h on an orbital shaker (100 rpm). To lyse the bacterial cells, overnight cultures were treated with 5% chloroform (v/v) for 30 min, and centrifuged at 12 000 × *g* for 15 min to remove cell debris. The phage lysates were filtered through a 0.2 μm bottle top filter (Millipore). Phage stocks were stored at 4°C in either 0.8% NB or 0.01 M phosphate buffer (PB, pH 6.8). The soft agar overlay method described by Adams (1959) was used to determine the concentration in pfu/ml of each phage stock solution.

### Multiplex real-time PCR

*Erwinia* spp. phages in the Vineland AAFC Collection were identified as *Myoviridae* (long contractile tail), *Siphoviridae* (long non-contractile tail) or *Podoviridae* (short tail) by restriction fragment length polymorphisms and electron microscopy (Gill *et al*., 2008). Specific real-time PCR primers and probes for *E. amylovora*, *P. agglomerans* (Lehman, 2007) and ones that target *Myoviridae* and *Podoviridae* specific genes were designed for multiplex PCR (Table 2). Design of real-time PCR primers and probes targeting *Siphoviridae* was not possible due to loss of these phage isolates from the collection as a result of long-term storage. Multiplex real-time PCR reactions comprised of 25 μl in either 10X ThermoPol buffer (NEB, Ipswich, MA), 0.2 mM of each primer, 0.1 mM of probe, 0.2 mM each of deoxyribonucleotide triphosphate (dNTP), 2 mM MgCl_2_, 1.3 U *Taq* polymerase (NEB) or 2X Master Mix (Qiagen) and 2 μl of sample template suspended in PB. The reaction was assayed on a Stratagene Mx4000 Quantitative PCR thermocycler (La Jolla, CA) (95°C, 5 min and 45 cycles of 95°C, 10 s, 60°C, 16 s) or a Corbett Rotor-Gene 6000 thermocycler (95°C, 5 min then 40 cycles of 95°C for 4 s and 60°C for 11 s). The threshold cycle (C_T_) was determined by machine software. Positive controls, samples with added phage, and no template controls were included with each test.

### Spontaneous liberation and induction of prophages from *P. agglomerans*

Two methods were used to test for the recovery of phage from bacteria, spontaneous liberation and induction. To assess the spontaneous release of phage particles, cells from an overnight culture in NB were collected by centrifugation at 12 000 × *g* for 4 min. For prophage induction, 5 μl aliquots from overnight cultures were diluted into 45 ml of NB and incubated until the population reached mid-log phase (OD_600_ 0.4). Cells were then treated with mitomycin C (MC; 0, 0.5 or 1 μg/ml) (Jiang and Paul, 1998) and incubated for an additional 18 h at 27°C before centrifugation at 12 000 × *g* for 4 min. Both spontaneous and induction treatments were assessed for phage particles by mixing the supernatant with exponentially growing sensitive bacterial cells, adding 3 ml of 0.8% NA and plating on 2.3% NA. After incubation for 24 h at 27°C, plates were examined for bacterial cell lysis.

### Isolation of bacteriophage insensitive mutants of *E. amylovora*

The agar plate method and the secondary culture method (Guglielmotti *et al*., 2006) were used to isolate BIMs. Bacterial cultures grown overnight in NB were inoculated with phages at MOI of 10, 1 or 0.1. Infected cells were mixed with soft agar, poured over 2.3% NA and incubated at 27°C for 24–48 h. Presumptive BIMs, appearing as single bacterial colonies, were isolated, cultured and tested for phage sensitivity. The secondary method was performed in a similar manner except after 24 h; streaks were taken from plates exhibiting no colony growth and were swabbed on fresh 2.3% NA medium for an additional 24 h at 27°C. Each BIM was subcultured four times on 2.3% NA in the absence of phages to confirm the stability of phage resistance. BIMs were stored at −80°C. In order to assess long-term stability, the frozen stock of each BIM was periodically sampled by streaking on NA plus 0.5% sucrose from and plates were incubated overnight at 27°C. For a final sample, 10 colonies per BIM stock were suspended individually in 100 μl of PB and tested with multiplex real-time PCR.

## Conflict of interest

None declared.

## Supporting Information

**Table S1.** Real-time PCR was used to confirm the identity of global wild-type isolates as *Erwinia amylovora*. Specific *Erwinia* spp. *Myoviridae* and *Podoviridae* probe-primers were used to screen the global population for the presence of prophages. **Table S2.** Isolate confirmation and lysogeny screening of wild-type *Pantoea agglomerans* isolated from field collected blossoms from southern Ontario, CA.
