# Supplementary material for: Absence of lysogeny in wild populations of Erwinia amylovora and Pantoea agglomerans
Source: Microb Biotechnol. 2015 Feb 12;8(3):510–8. doi: 10.1111/1751-7915.12253 (PMC4408183; doi:10.1111/1751-7915.12253)
Supplement: Supplementary file 1 [file mbt20008-0510-sd1.doc]

**Supplement 1**. Real time PCR was used to confirm the identity of global wild typeisolates as*Erwinia amylovora*. Specific *Erwinia* spp. *Myoviridae* and *Podoviridae* probe-primers were used to screen the globalpopulationfor the presence of prophages.

|  | **Isolate** | | ***Source*** | **real time PCR diagnosis** | | |  | Prophage Inductionwith  mitomycin C ^c^ |
| --- | --- | --- | --- | --- | --- | --- | --- | --- |
|  |  |  |  | ***E. amylovora***^a^ | ***P. agglomerans***^a^ | **Prophage**^b^ |  |  |
| ***E. amylovora*** | | | |  |  |  |  |  |
| Canada | | |  |  |  |  |  |  |
|  | Ontario^d^ | |  |  |  |  |  |  |
|  |  | Ea6-4 | apple | *+* | - | - |  | - |
|  |  | EaD-7 | apple | *+* | - | - |  | - |
|  |  | EaG-5 | pear | *+* | - | - |  | - |
|  |  | Ea17-1-1 | apple | *+* | - | - |  | - |
|  |  | Ea29-7 | pear | *+* | - | - |  | - |
|  | British Columbia^e^ | |  |  |  |  |  |  |
|  |  | Ea20A | crab apple | + |  | - |  | NT |
|  |  | Ea21 | nanking cherry | + | - | - |  | NT |
|  |  | Ea23A | edge wood | + | - | - |  | NT |
|  |  | Ea29 | pear | + | - | - |  | NT |
|  |  | Ea31B | pear | + | - | - |  | NT |
|  |  | Ea34A | apple | + | - | - |  | NT |
|  |  | Ea5 | apple | + | - | - |  | NT |
|  |  | Ea1280 | apple | + | - | - |  | NT |
|  |  | Ea1337 | apple | + | - | - |  | NT |
|  |  | Ea2345 | apple | + | - | - |  | NT |
|  | Nova Scotia^f^ | | |  |  |  |  |  |
|  |  | Ea1-95 | raspberry | + | - | - |  | NT |
|  |  | Ea1-97 | raspberry | + | - | - |  | NT |
|  |  | Ea2-95 | raspberry | + | - | - |  | NT |
|  |  | Ea2-97 | raspberry | + | - | - |  | NT |
|  |  | Ea3-97 | raspberry | + | - | - |  | NT |
|  |  | Ea4-96 | raspberry | + | - | - |  | NT |
|  |  | Ea5-96b | raspberry | + | - | - |  | NT |
|  |  | Ea6-96b | raspberry | + | - | - |  | NT |
|  |  | Ea7-96b | raspberry | + | - | - |  | NT |
|  |  | Ea8-96 | raspberry | + | - | - |  | NT |
|  | United States | | |  |  |  |  |  |
|  | California^g^ | |  |  |  |  |  |  |
|  |  | Ea Apl 1 | apple | *+* | - | - |  |  |
|  |  | Ea lf | apple | *+* | - | - |  | NT |
|  |  | Ea7a | pear | *+* | - | - |  | NT |
|  |  | Ea8 | pear | *+* | - | - |  | NT |
|  |  | Ea12 | pear | *+* | - | - |  | NT |
|  |  | Ea21 | pear | *+* | - | - |  | NT |
|  |  | Ea43 | pear | *+* | - | - |  | NT |
|  |  | Ea60 | pear | *+* | - | - |  | NT |
|  |  | Ea91 | pear | *+* | - | - |  | NT |
|  |  | Ea120 | pear | *+* | - | - |  | NT |
|  |  | Ea168 | pear | *+* | - | - |  |  |
|  |  | Ea176 | pear | *+* | - | - |  | NT |
|  |  | Ea184 | pear | *+* | - | - |  | NT |
|  |  | Ea205 | pear | *+* | - | - |  | NT |
|  | Michigan^h^ | |  |  |  |  |  |  |
|  |  | BB-1 | apple | *+* | - | - |  | NT |
|  |  | D-1 | apple | *+* | - | - |  | NT |
|  |  | Ea110^i^ | apple | *+* | - | - |  | - |
|  |  | EL01 | apple | *+* | - | - |  | NT |
|  |  | GM-1 | apple | *+* | - | - |  | NT |
|  |  | MK1 | apple | *+* | - | - |  | NT |
|  |  | MI 6-2 | apple | *+* | - | - |  | NT |
|  |  | MI 14-2 | apple | *+* | - | - |  | NT |
|  |  | P-C-1 | apple | *+* | - | - |  | NT |
|  |  | RB02 | apple | *+* | - | - |  | NT |
|  |  | RB07 | apple | *+* | - | - |  | NT |
|  |  | RN8 | apple | *+* | - | - |  | NT |
|  |  | Root C-2 | apple | *+* | - | - |  | NT |
|  |  | SL | apple | *+* | - | - |  | NT |
|  |  | T-1 | apple | *+* | - | - |  | NT |
|  |  | BJN | apple | *+* | - | - |  | NT |
|  | New York^j,k^ | | |  |  |  |  |  |
|  |  | 100-1 | unknown | + | - | - |  | NT |
|  |  | 110-1 | unknown | + | - | - |  | NT |
|  |  | C-22 | unknown | + | - | - |  | NT |
|  |  | C-GG-11 | apple | + | - | - |  | NT |
|  |  | Ea273 | apple | + | - | - |  | NT |
|  |  | O-TO-1b | apple | + | - | - |  | NT |
|  |  | O-RG-11 | apple | + | - | - |  | NT |
|  |  | O-RG-21 | apple | + | - | - |  | NT |
|  |  | P-C-3b | apple | + | - | - |  | NT |
|  |  | T-RG-1-1 | apple | + | - | - |  | NT |
|  |  | T-RG-5a | apple | + | - | - |  | NT |
|  | Oregon^l^ | | |  |  |  |  |  |
|  |  | ATCC 51852 | plum | + | - | - |  | NT |
|  |  | ATCC 51853 | plum | + | - | - |  | NT |
|  |  | LA469 (Ea138) | apple | + | - | - |  | NT |
|  |  | LA470 (Ea144 ) | apple | + | - | - |  | NT |
|  |  | LA471 (HR7) | pear | + | - | - |  | NT |
|  |  | LA472 (HR11) | pear | + | - | - |  | NT |
|  |  | LA473 | pear | + | - | - |  | NT |
|  |  | LA474 | pear | + | - | - |  | NT |
|  |  | LA475 | pear | + | - | - |  | NT |
|  |  | LA476 | pear | + | - | - |  | NT |
|  |  | LA477 | pear | + | - | - |  | NT |
|  |  | LA478 | pear | + | - | - |  | NT |
|  | France^m^ | | |  |  |  |  |  |
|  |  | F1 | hawthorn | *+* | - | - |  | NT |
|  |  | F2 | apple | *+* | - | - |  | NT |
|  |  | F3 | apple | *+* | - | - |  | NT |
|  |  | F4 | apple | *+* | - | - |  | NT |
|  |  | F5 | apple | *+* | - | - |  | NT |
|  |  | F6 | apple | *+* | - | - |  | NT |
|  |  | F7 | apple | *+* | - | - |  | NT |
|  |  | F8 | apple | *+* | - | - |  | NT |
|  |  | F9 | apple | *+* | - | - |  | NT |
|  |  | F10 | apple | *+* | - | - |  | NT |
|  | Germany^n^ | | |  |  |  |  |  |
|  |  | 213/07 | apple | *+* | - | - |  | NT |
|  |  | 221/07 | quince | *+* | - | - |  | NT |
|  |  | 224/07 | quince | *+* | - | - |  | NT |
|  |  | 227/07 | mountain ash | *+* | - | - |  | NT |
|  |  | 234/07 | apple | *+* | - | - |  | NT |
|  |  | 243/07 | cotoneaster | *+* | - | - |  | NT |
|  |  | 245 | apple | *+* | - | - |  | NT |
|  |  | 250/07 | apple | *+* | - | - |  | NT |
|  |  | 280 | apple | *+* | - | - |  | NT |
|  |  | 294 | apple | *+* | - | - |  | NT |
|  | Israel^o^ | | |  |  |  |  |  |
|  |  | Ea169 | pear | + | - | - |  | NT |
|  |  | Ea209 | pear | + | - | - |  | NT |
|  |  | Ea238 | pear | + | - | - |  | NT |
|  |  | Ea241 | pear | + | - | - |  | NT |
|  |  | Ea249 | pear | + | - | - |  | NT |
|  |  | Ea276 | pear | + | - | - |  | NT |
|  |  | Ea308 | pear | + | - | - |  | NT |
|  |  | Ea321 | pear | + | - | - |  | NT |
|  |  | Ea328 | pear | + | - | - |  | NT |
|  |  | Ea401 | pear | + | - | - |  | NT |
|  | Lebanon^h^ | | |  |  |  |  |  |
|  |  | A7 | quince | + | - | - |  | NT |
|  |  | A-11 | apple | + | - | - |  | NT |
|  |  | A-22 | apple | + | - | - |  | NT |
|  |  | A-24 | apple | + | - | - |  | NT |
|  |  | B-3 | apple | + | - | - |  | NT |
|  |  | B-51 | apple | + | - | - |  | NT |
|  |  | B-58 | apple | + | - | - |  | NT |
|  |  | B-65 | apple | + | - | - |  | NT |
|  |  | B-95 | apple | + | - | - |  | NT |
|  |  | B-110 | apple | + | - | - |  | NT |
|  |  | Leb B66 | unknown | + | - | - |  | NT |
|  |  | Leb BB66 | unknown | + | - | - |  | NT |
|  | Morocco^m^ | | |  |  |  |  |  |
|  |  | M1 | unknown | *+* | - | - |  | NT |
|  |  | M2 | unknown | *+* | - | - |  | NT |
|  |  | M3 | unknown | *+* | - | - |  | NT |
|  |  | M4 | unknown | *+* | - | - |  | NT |
|  |  | M5 | unknown | *+* | - | - |  | NT |
|  |  | M6 | unknown | *+* | - | - |  | NT |
|  |  | M7 | unknown | *+* | - | - |  | NT |
|  |  | M8 | unknown | *+* | - | - |  | NT |
|  |  | M9 | unknown | *+* | - | - |  | NT |
|  |  | M10 | unknown | *+* | - | - |  | NT |
|  | New Zealand^p^ | | |  |  |  |  |  |
|  |  | Ea 315-1 | apple | *+* | - | - |  | NT |
|  |  | Ea 412-1 | pear | *+* | - | - |  | NT |
|  |  | Ea 501 | unknown | *+* | - | - |  | NT |
|  |  | FB 29 | pear | *+* | - | - |  | NT |
|  |  | FB 78a | apple | *+* | - | - |  | NT |
|  |  | FB 97b | apple | *+* | - | - |  | NT |
|  | Poland^q^ | |  |  |  |  |  |  |
|  |  | 367 | firethorn | + | - | - |  | NT |
|  |  | 462 | apple | + | - | - |  | NT |
|  |  | 464 | pear | + | - | - |  | NT |
|  |  | 614a | pear | + | - | - |  | NT |
|  |  | 616a | apple | + | - | - |  | NT |
|  |  | 624a | pear | + | - | - |  | NT |
|  |  | 633a | apple | + | - | - |  | NT |
|  |  | 650 | hawthorn | + | - | - |  | NT |
|  |  | 660 | pear | + | - | - |  | NT |
|  |  | 661 | mountain ash | + | - | - |  | NT |
|  |  | 694 | apple | + | - | - |  | NT |
|  |  | E-10 | apple | + | - | - |  | NT |
|  | Spain^r^ | |  |  |  |  |  |  |
|  |  | IVIA 1526-1 | cotoneaster | *+* | - | - |  | NT |
|  |  | IVIA 1739-1 | apple | *+* | - | - |  | NT |
|  |  | IVIA 1777-1 | firethorn | *+* | - | - |  | NT |
|  |  | IVIA 1892-1 | pear | *+* | - | - |  | NT |
|  |  | IVIA 1924-4 | firethorn | *+* | - | - |  | NT |
|  |  | IVIA 2303-3 | pear | *+* | - | - |  | NT |
|  |  | IVIA 2311-6 | pear | *+* | - | - |  | NT |
|  |  | IVIA 2349-2 | pear | *+* | - | - |  | NT |
|  |  | IVIA 2397-5.4 | pear | *+* | - | - |  | NT |
|  | Switzerland^s^ | |  |  |  |  |  |  |
|  |  | ACW 35260 | hawthorn | + | - | - |  | NT |
|  |  | ACW 42107 | pear | + | - | - |  | NT |
|  |  | ACW 42287 | cotoneaster | + | - | - |  | NT |
|  |  | ACW 42288 | pear | + | - | - |  | NT |
|  |  |  |  |  |  |  |  |  |
|  |  |  |  |  |  |  |  |  |

^a^ Isolate identity confirmed using real-time PCR probe/primers (table 2), + indicates successful amplification, - indicates no amplification.

^b^ Presence of prophage tested through real-time PCR, utilizing STS3 (*Podoviridae*) and Pun45 (*Myoviridae*) probe/primer sets, + indicates successful amplification, - indicates no amplification.

^c^ Induction of prophage lytic cycle with mitomicin C, determined by plaque formation on 6 indicator isolates, + indicates plaque formation, - indicates no plaque formation

^d^ A.M. Svircev, Southern Crop Protection and Food Research Centre, Agriculture and Agri-Food Canada, Vineland Station, Ontario, Canada L0R 2E0

^e^ Peter Sholberg and Julie Boulé,Pacific Agri-Food Research Centre, Agriculture and Agri-Food Canada, 4200 Highway 97, Summerland, B.C., CanadaV0H 1Z0

^f^ P.G. Braun, Atlantic Food and Horticulture Research Centre, Agriculture and Agri-Food Canada, 32 Main Street Kentville, NS, Canada B4N 1J5

^g^ Steven E. Lindow, Department of Plant and Microbial Biology, University of California, Berkeley, California, USA 94720

^h^George Sundin, Michigan State University, 578 Wilson, East Lansing, Michigan, USA 48824

^i^Al Jones, Department of Plant Pathology, Michigan State University, East Lansing, Michigan, USA. 48824-1312

^j^Herb S. Aldwinckle, Department of Horticultural Sciences, Cornell University, New York StateAgricultural Experiment Station, Geneva, NY, USA 14456

^k^Steven V. Beer, Department of Plant Pathology, Cornell University, Ithaca, NY, USA 14853

^l^Virginia Stockwell, Department of Botany and Plant Pathology, Oregon State University, Corvallis, Oregon, USA 97330

^m^Jean-Pierre Paulin, Institut national de la recherche agronomique, 3INRA, Station de Pathologie Végétale, 42 rue Georges Morel, B.P. 57, Beaucouzé, France49071.

^n^ Esther Moltmann, Landesanstalt für Pflanzenschutz, Reinsburgstrase 107, Stuttgart, Germany D-70197

^o^ S. Manulis­­­­­­­­­­­­­­­­, Department of Plant Pathology, Agriculture Research Organization, The Volcani Center­, Bet Dagan, Israel 1048-E.

^p^Joel Vanneste, Ruakura Research Centre, East Street,Hamilton, New Zealand3214

^q^Piotr Sobiczewski, Research Institute of Pomology and Floriculture, Pomologiczna 18, Skierniewice, Poland 96-100

^r^  Maria Lopez, Instituto Valenciano de Investigaciones Agrarias, Valencia, Spain.

^s^ Brion Duffy, Agroscope Changins-Wädenswil ACW, Plant Protection Division, CH-8820 Wädenswil, Switzerland.

**Supplement 2.** Isolate confirmation and lysogeny screening of wild type *Pantoea agglomerans* isolated from field collected blossoms from southern Ontario, CA.

|  | | Colony Morphology ^a^ | | PA Confirmation ^b^ | Prophage Presence^c^ | Prophage^d^ |
| --- | --- | --- | --- | --- | --- | --- |
| Isolate | Source^e^ | MMS | NA | (real time PCR) | (real time PCR) | (Spontaneous release) |
| Eh 1 | unknown | NT | Y | + | - | - |
| Eh 1-28a | apple | NT | W | + | - | - |
| Eh 1-28b | apple | NT | Y | + | - | - |
| Eh 17-17 | apple | NT | W | + | - | - |
| Eh 21-5 | pear | NT | Y | + | - | - |
| Eh 7-5 | unknown | NT | W | + | - | - |
| Pa2-2 | pear | O | Y | + | - | - |
| Pa9-1 | apple | O | Y | + | - | - |
| Pa9-2 | apple | O | W | + | - | - |
| Pa9-4 | apple | O | W | + | - | - |
| Pa13-1 | pear | O | Y | + | - | - |
| Pa13-2 | pear | O | Y | + | - | - |
| Pa13-3 | pear | O | Y | + | - | - |
| Pa13-4 | pear | O | Y | + | - | - |
| Pa13-5 | pear | O | Y | + | - | - |
| Pa17-1 | pear | O | Y | + | - | - |
| Pa17-2 | pear | O | Y | + | - | - |
| Pa17-3 | pear | O | Y | + | - | - |
| Pa17-4 | pear | O | Y | + | - | - |
| Pa17-5 | pear | O | Y | + | - | - |
| Pa21-2 | pear | O | Y | + | - | - |
| Pa21-3 | pear | O | Y | + | - | - |
| Pa21-4 | pear | O | Y | + | - | - |
| Pa21-5 | pear | O | W | + | - | - |
| Pa21-6 | pear | O | Y | + | - | - |
| Pa21-7 | pear | O | W | + | - | - |
| Pa21-9 | pear | O | Y | + | - | - |
| Pa21-10 | pear | O | Y | + | - | - |
| Pa21-11 | pear | O | W | + | - | - |
| Pa21-12 | pear | O | Y | + | - | - |
| Pa21-13 | pear | O | Y | + | - | - |
| Pa21-14 | pear | O | Y | + | - | - |
| Pa21-15 | pear | O | Y | + | - | - |
| Pa21-16 | pear | O | Y | + | - | - |
| Pa21-17 | pear | O | Y | + | - | - |
| Pa21-18 | pear | O | Y | + | - | - |
| Pa21-19 | pear | O | Y | + | - | - |
| Pa21-20 | pear | O | Y | + | - | - |
| Pa21-21 | pear | O | Y | + | - | - |
| Pa31-1 | apple | O | Y | + | - | - |
| Pa31-2 | apple | O | Y | + | - | - |
| Pa31-3 | apple | O | Y | + | - | - |
| Pa31-5 | apple | O | Y | + | - | - |
| Pa31-6 | apple | O | Y | + | - | - |
| Pa31-7 | apple | O | Y | + | - | - |
| Pa31-8 | apple | O | Y | + | - | - |
| Pa39-1 | mountain ash | O | Y | + | - | - |
| Pa39-2 | mountain ash | O | Y | + | - | - |
| Pa39-3 | crab apple | O | Y | + | - | - |
| Pa39-4 | crab apple | O | Y | + | - | - |
| Pa39-5 | crab apple | O | Y | + | - | - |
| Pa39-6 | crab apple | O | Y | + | - | - |
| Pa39-7 | mountain ash | O | Y | + | - | - |
| Pa39-8 | crab apple | O | Y | + | - | - |
| Pa39-9 | crab apple | O | Y | + | - | - |
| Pa39-10 | mountain ash | O | Y | + | - | - |
| Pa39-11 | crab apple | O | Y | + | - | - |
| Pa39-28 | crab apple | O | Y | + | - | - |
| Pa39-12 | hawthorn | O | Y | + | - | - |
| Pa39-13 | apple | O | Y | + | - | - |
| Pa39-29 | hawthorn | O | Y | + | - | - |
| Pa39-14 | hawthorn | O | Y | + | - | - |
| Pa39-15 | hawthorn | O | Y | + | - | - |
| Pa39-16 | hawthorn | O | Y | + | - | - |
| Pa39-17 | hawthorn | O | Y | + | - | - |
| Pa39-18 | hawthorn | O | Y | + | - | - |
| Pa39-19 | crab apple | O | Y | + | - | - |
| Pa39-20 | cotoneaster | O | Y | + | - | - |
| Pa39-21 | hawthorn | O | Y | + | - | - |
| Pa39-22 | hawthorn | O | Y | + | - | - |
| Pa39-23 | crab apple | O | Y | + | - | - |
| Pa39-26 | apple | O | Y | + | - | - |
| Pa39-24 | mountain ash | O | Y | + | - | - |
| Pa39-25 | mountain ash | O | Y | + | - | - |
| Pa39-27 | crab apple | O | Y | + | - | - |
| Pa39-30 | crab apple | O | Y | + | - | - |
| Pa45-1 | unknown | O | Y | + | - | - |
| Pa45-2 | unknown | O | Y | + | - | - |
| Pa45-3 | unknown | O | Y | + | - | - |
| Pa46-2 | crab apple | O | Y | + | - | - |
| Pa49-1 | pear | O | Y | + | - | - |
| Pa-HJ | unknown | O | Y | + | - | - |

^a^ Isolate identity confirmed through appearance on MMS (Modified Miller-Schroth Agar) and NA (Nutrient Agar, Difco), NT-Not tested, O-Orange, W-White, Y-Yellow.

^b^ Isolate confirmation using real-time PCR, Pa-gnd probe/primer, + indicates successful amplification, - indicates no amplification.

^c^ Presence of prophage tested through real-time PCR, utilizing STS3 (*Podoviridae*) and Pun45 (*Myoviridae*) probe/Primer sets, + indicates successful amplification, - indicates no amplification.

^d^Culture supernatants tested for spontaneous release of phages,determined by plaque formation on 6 indicator isolates, + indicates plaque formation, - indicates no plaque formation .

^e^ Isolates from southern Ontario, A.M. Svircev, Southern Crop Protection and Food Research Centre, Agriculture and Agri-Food Canada, Vineland Station, Ontario, Canada L0R 2E0
